# Effect of Textured Dimples on the Tribological Behavior of WC/Co Cemented Carbide in Dry Sliding with Al_2_O_3_/WC Ceramic

**DOI:** 10.3390/mi13081269

**Published:** 2022-08-06

**Authors:** Jiansong Chen, Ze Wu

**Affiliations:** School of Mechanical Engineering, Southeast University, Nanjing 211189, China

**Keywords:** surface texturing, cemented carbide, ceramic, friction, wear

## Abstract

Micro-dimples were fabricated on the surface of WC/Co cemented carbide disks by laser, and dry friction tests were carried out by sliding with Al_2_O_3_/WC ceramic balls. Results show that the textured cemented carbide can reduce the average friction coefficient by about 30% compared to the smooth sample, while the textured cemented carbide with solid lubricants can reduce the average friction coefficient by about 50%. The density of textured dimples has no obvious influence on the friction coefficient. The wear rates of worn ceramic balls continue to decline with the increase in sliding speeds. The wear rates of the ceramic balls can be reduced by 40~50% for textured samples and about 65% for textured samples with solid lubricants compared to the untextured ones. The mechanism for improving the tribological properties of cemented carbide materials is that the textured dimples can store lubricants and capture wear debris, which would play an important role in promoting the engineering application of surface texturing in cemented carbide materials.

## 1. Introduction

Due to the increasing application requirements of functional materials, surface texturing technology has been paid enough attention. In recent decades, surface texturing has become a critical area in material engineering for its superior ability to control the properties of an interface. It has been confirmed that surface texturing can enhance performance in different tribological environments such as abrasive wear, adhesive wear, wear with solid lubrication, as well as hydrodynamic lubrication [1]. It is also confirmed that surface texturing can not only improve tribological performance, can help in obtaining high-hydrophobic coatings [2,3], and can improve biological implants [4,5] and the wettability of materials [6]. Geometries and sizes of textured surfaces have important influences on their tribological applications. The effects of parameters such as shapes [7,8,9], spacing [10], width [11], and area fractions [12] of surface texturing on tribological performances have been investigated.

At present, the research for tribological application of surface texturing mainly focuses on ceramic materials and metal materials [13,14]. For example, Xing et al. [15], Ji et al. [16], Kumar et al. [17], Yusuf et al. [18] and Vedha Hari et al. [19] investigate the tribological properties of surface texturing on metal oxide ceramics. Xing et al. [15] indicate that a combination of laser surface textures and multiple solid lubricant coatings effectively improves the tribological properties of Al_2_O_3_/TiC ceramic. Ji et al. [16] fabricate micro-dimple, micro-groove and micro-grid textures onto dental zirconia ceramic, and verify the surface texturing has an important effect on the wettability of zirconia ceramic by modifying the contact condition between the liquid and the solid. It is reported by Kumar et al. [17] that the direction of micro-grooves on the Al_2_O_3_/TiCN ceramic tool has a key impact on tool wear. Yusuf et al. [18] prove that the presence of textured dimples on the surface affects the antibacterial properties of the TiO_2_/ZnO coatings. Vedha Hari et al. [19] indicate that textured alumina ceramic tools can play to superior performance in dry cutting of grey cast iron. Xing et al. [20] and Liu et al. [21] investigate the tribological properties of surface texturing on silicon nitride ceramics. It is also proved by Xing et al. [20] that laser-dimpled textures combined with DLC coatings on Si_3_N_4_/TiC ceramic have excellent tribological properties. Liu et al. [21] indicate that laser surface texturing improves the flexural strength of Si_3_N_4_ ceramic for it reduces the effective volume and effective surface remarkably.

Metal materials have good adaptability to surface texture. For example, Liu et al. [22] report that high surface roughness and high specific area induced by laser surface texturing can promote the deposition of phosphate conversion coating on magnesium alloy. Zhang et al. [23] indicate that optimized surface texturing on 42CrMo matrix composite can not only reduce the friction coefficient and wear rate but also enhance the stability of the friction system. It is proved by Yuan et al. [24] that surface texturing plays a positive role in improving the wear resistance of four kinds of metal materials (316 SS, NiTi, TA2, and Ti6Al4V), especially under oil lubrication conditions. Li et al. [25] report that the wettability of a 4043 aluminum alloy on 301L stainless steel can be enhanced by chemical-etched surface texturing. Dai et al. [26] indicate that masked laser surface texturing and hard contact laser shock peening for Ti6Al4V surface can provide good friction and wear resistance performance. It is also reported by Wei et al. [27] that the micro-dimpled texture on AISI 52,100 bearing steel could reduce friction coefficient under dry and lubricated conditions.

At present, the research for tribological application of surface texturing on cemented carbide material is relatively few. For example, He et al. [28] propose a research method based on the synergistic effect of laser process parameters and texture arrangement parameters on the surface friction properties of micro-textured cemented carbide, which provides a theoretical basis for further optimization and development of micro-textured tools.

According to the above statement, it can be seen that surface texturing plays an important role in improving tribological performance for ceramic materials and metal materials. Cemented carbide is a typical hard brittle material, which has a wide range of applications in engineering. The tribological studies on surface texturing of cemented carbide are relatively few. In the present study, micro-dimpled texturing for WC/Co cemented carbide is proposed. The tribological performance of the textured cemented carbide samples in dry sliding with Al_2_O_3_/WC ceramic is investigated. The study would have potential value for promoting the engineering application of surface texturing in cemented carbide.

## 2. Experimental Procedures

### 2.1. Materials

The selected specimens were commercial hot pressed sintered cemented carbide discs with a diameter of 56 mm and a thickness of 4 mm. The composition of the cemented carbide consisted of 6 wt% cobalt (Co) and wolfram carbide (WC) with a grain size of 0.8~1.5 µm. The WC was a hard phase, while the Co was a bonding phase. The properties of the WC/Co cemented carbide are listed in Table 1. The friction material used was a ceramic ball with a diameter of 9.525 mm. The composition of the ceramic consisted of 10 wt% cobalt (Co), 35 wt% wolfram carbide (WC) and aluminum oxide (Al_2_O_3_). The properties of the Al_2_O_3_/WC ceramic are listed in Table 2.

### 2.2. Preparation of Textured Dimples

**Figure 1:**
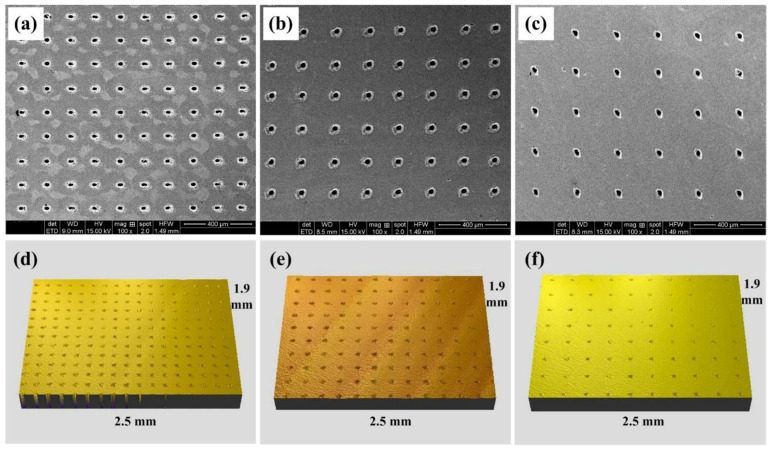
Morphology of textured dimples: (**a**) TD150, (**b**) TD200 and (**c**) TD250, as well as the corresponding stereoscopic topography: (**d**) 3D TD150, (**e**) 3D TD200 and (**f**) 3D TD250.

**Figure 2:**
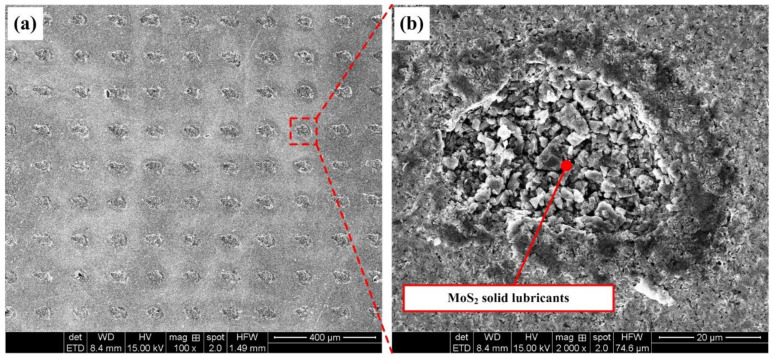
Morphology of textured dimples filled with MoS_2_ solid lubricants: (**a**) TD150 filled with solid lubricants and (**b**) enlarged view of a single dimple.

The upper surfaces of the WC/Co cemented carbide disks were polished with a diamond polishing agent to obtain a roughness of 0.03 μm. Micro-dimple patterns were then created on the upper surface of the cemented carbide disks by an LD side-pumped solid-state laser. The laser pulse was generated using an Nd: YAG laser system at a center wavelength of 1064 nm, a repetition rate of 2 kHz and a pulse duration of about 20 ns. Machining was accomplished in air with the average operating voltage of 10 V, the average laser power of 20 W, the laser spot diameter of 15 µm and the focusing length of 80 mm. After laser texturing, a gentle polishing process was carried out to remove bulges or burrs around the rim of the dimples. After one minute of polishing with the diamond polishing agent, bulges or burrs around the rim of the dimples were completely removed. The diameter of a single dimple is about 40 μm, while the depth is about 50 μm. Three kinds of textured patterns with dimple-distance of 150 μm (TD150), 200 μm (TD200) and 250 μm (TD250) were designed and fabricated, respectively. The textured patterns with different dimple-distance on the surface of cemented carbides are shown in Figure 1a–c, while the corresponding stereoscopic topography are presented in Figure 1d–f. Some of the TD150 samples were selected to fill molybdenum disulfide (MoS_2_) solid lubricants in the textured dimples. MoS_2_ is gray and slightly blue in appearance, with a sense of greasiness, and it has a layered hexagonal crystal structure, with a density of 4.5 g/cm^3^ and a melting point of 1185 °C. The morphology of textured dimples filled with MoS_2_ solid lubricants is shown in Figure 2.

### 2.3. Friction and Wear Test

**Figure 3:**
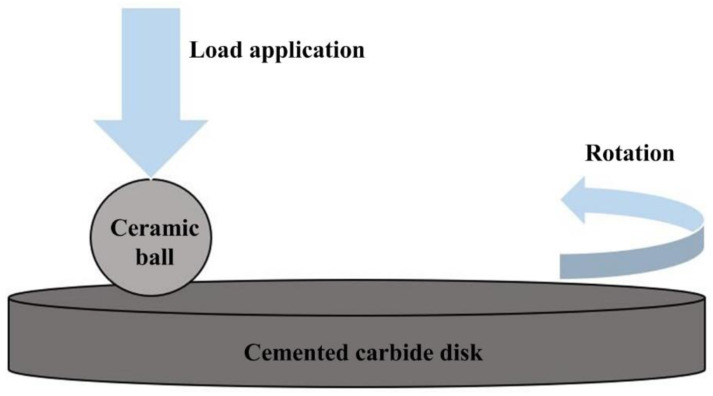
Schematic diagram of the friction and wear test.

Rotating ball-on-disk tests were carried out using a tribometer (UMT-2, USA) to investigate the tribological performance of dimple-textured WC/Co cemented carbide samples sliding against Al_2_O_3_/WC ceramic balls. The schematic diagram of the friction and wear test is shown in Figure 3. The ceramic ball was fixed, while the cemented carbide disk was rotated. Friction and wear tests were conducted with the sliding speeds varied from 20 m/min to 100 m/min, a fixed time of 10 min and an applied load of 10 N. The friction coefficient for smooth sample (SS), TD150 with and without solid lubricants, TD200 and TD250 samples were tested for comparison. When it was found that the texture density had no obvious effect on the friction coefficient, the analysis of wear loss of ceramic balls is only for comparison with the smooth sample (SS), TD150 with and without solid lubricants.

The topography of the worn cemented carbide disks as well as the ceramic balls were studied using a scanning electron microscope (SEM) and an energy dispersive X-ray analysis (EDS). The wear loss of the ceramic balls was calculated based on the wear marks, which were regarded as a spherical crown. As a result, the specific wear rate was derived as the ratio of wear volume loss over the applied load multiplied by the total sliding distance. The mechanism for the tribological performance of the surface texture on the cemented carbide was discussed based on microscopic detection and analysis.

## 3. Results and Discussion

### 3.1. Friction Coefficient

**Figure 4:**
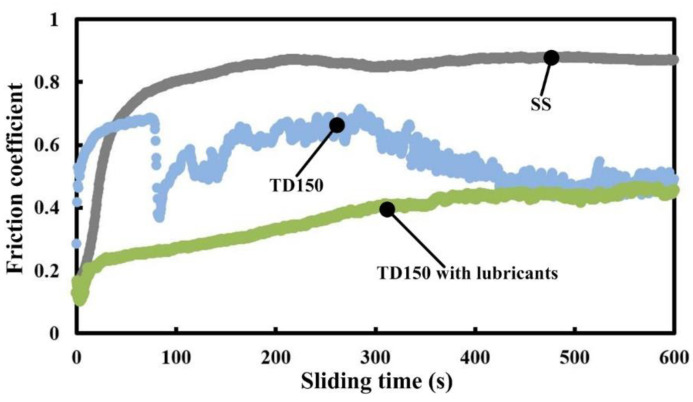
Variations of friction coefficient of different samples at sliding speed of 40 m/min.

The variations of friction coefficient of three kinds of samples (SS, TD150 and TD150 with lubricants) at a sliding speed of 40 m/min are presented in Figure 4. The friction coefficient for the SS sample first increases and then maintains a relatively stable value. The friction coefficient for the TD150 sample rapidly increases and then drops to a smaller value with obvious fluctuation. However, a small and slowly increasing friction coefficient is obtained in the operation by TD150 sample with lubricants. After sliding friction for 7 min, the values of friction coefficients for TD150 with or without lubricants tend to be consistent. In general, the friction coefficient for TD150 with lubricants is the smallest and has no obvious fluctuation.

**Figure 5:**
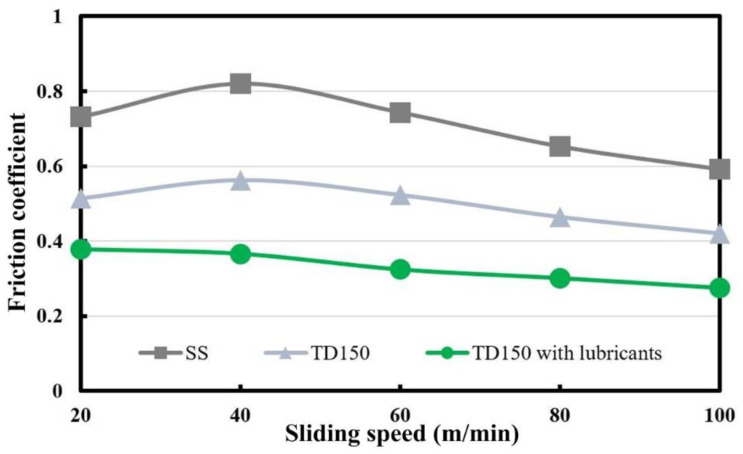
Average friction coefficients of different samples at different sliding speeds.

The average value of the fluctuant friction coefficient obtained in friction operation was calculated and taken as the average friction coefficient for each sliding process. The average friction coefficients of three kinds of samples (SS, TD150 and TD150 with lubricants) at different sliding speeds are presented in Figure 5. It can be seen that the average friction coefficient increases with the increase of sliding speed to a maximum value of 0.82 for the SS sample, 0.563 for the TD150 sample achieved at the sliding speed of 40 m/min, and then continues to decline with the increase of sliding speed. The difference is that the average friction coefficient for the TD150 sample with solid lubricants continues to decline as the sliding speed changes from 20 m/min to 100 m/min. It can be found by a simple calculation from Figure 5 that the average friction coefficient for the TD150 sample is reduced by about 30% compared to that for the SS one, while the value for the TD150 sample with solid lubricants is reduced by about 50%.

**Figure 6:**
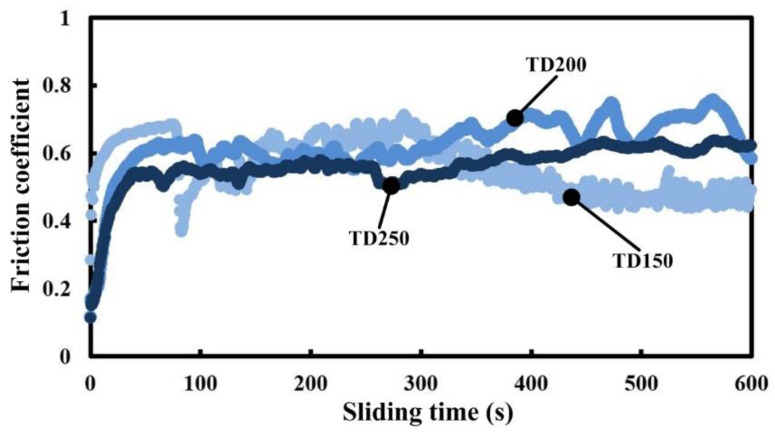
Variations of friction coefficient of different textured dimple samples at sliding speed of 40 m/min.

In order to investigate the effect of the density of micro-dimples on the tribological properties of cemented carbide materials, friction tests for samples with different texture parameters were also carried out. Figure 6 shows the friction coefficient curves of three different patterns (TD150, TD200 and TD250) at the sliding speed of 40 m/min. The friction coefficients for three different patterns (TD150, TD200 and TD250) all rapidly increase and then drop to a relatively stable value with obvious fluctuation. A significant difference is not found between the three different patterns. In other words, the density of dimples has no significant effect on the tribological properties of cemented carbide under current experimental conditions.

### 3.2. Wear Loss of the Ceramic Ball

In this research work, the hardness of the ceramic ball is slightly less than that of cemented carbide disk as presented in Table 1 and Table 2. As a result, the wear mode is mainly the wear of ceramic balls, while the surface wear on cemented carbide discs is very slight. Therefore, only the wear of cemented carbide balls is analyzed in this work.

**Figure 7:**
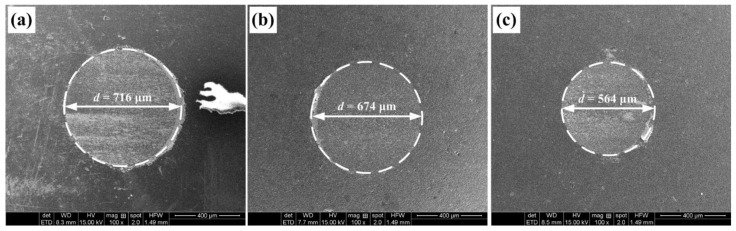
SEM images of the worn surface of the ceramic balls sliding with (**a**) SS, (**b**) TD150 and (**c**) TD150 with lubricants after 10 min dry operation at the speed of 40 m/min.

The SEM images of the worn surface of the ceramic balls after 10 min dr y sliding at the speed of 40 m/min are presented in Figure 7. The diameter of wear marks of the ceramic balls sliding with SS sample, TD150 sample and TD150 sample with solid lubricants are 716 µm, 674 µm and 564 µm, respectively.

**Figure 8:**
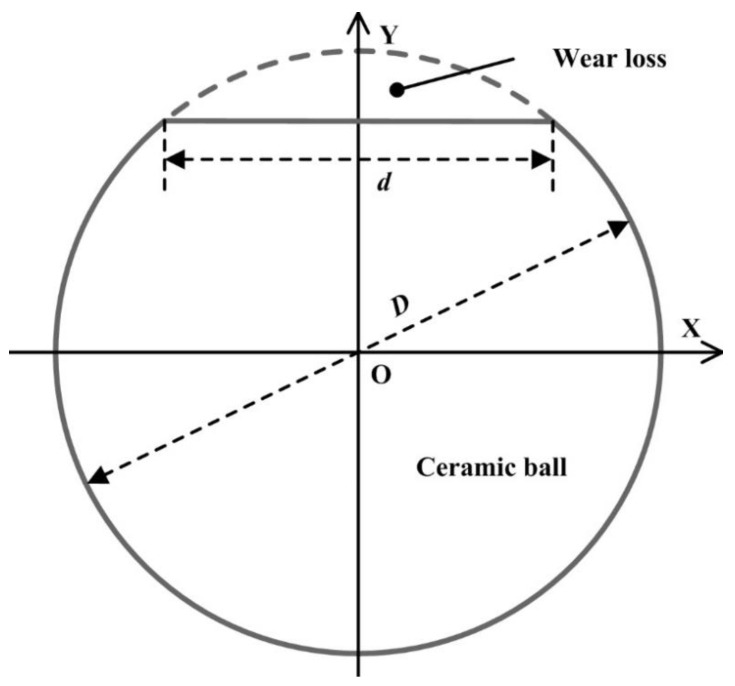
Schematic diagram of wear loss for the worn ceramic ball.

The schematic diagram of wear loss for the worn ceramic ball is shown in Figure 8. The wear loss of the ceramic ball can be regarded as a spherical crown, and the wear volume of the worn ball can be calculated as

(1) $$V=\int_{\frac{\sqrt{D^{2}-d^{2}}}{2}}^{\frac{D}{2}}\pi \left(\frac{D^{2}}{4}-y^{2}\right)dy=\frac{1}{12}\pi D^{3}-\frac{1}{24}\left(2\pi D^{2}+\pi d^{2}\right)\sqrt{D^{2}-d^{2}}$$

where *D* is the diameter of the ceramic ball, and *d* is the diameter of the wear mark.

**Figure 9:**
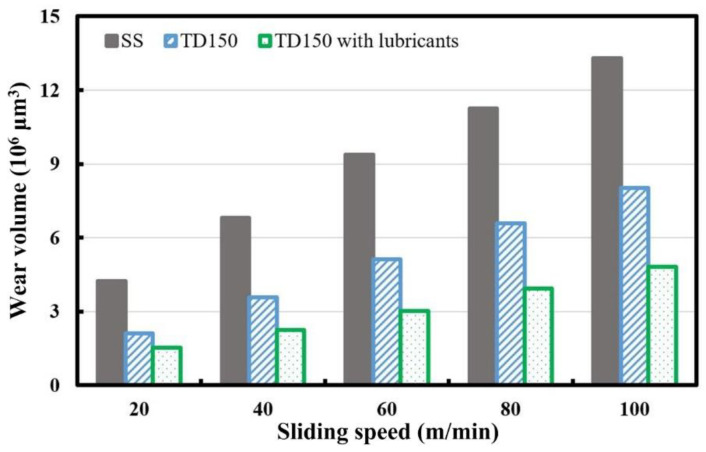
Wear volumes of the worn ceramic balls sliding with different samples at different speeds.

The wear volumes of the worn ceramic balls are calculated according to Equation (1). The wear volumes of the worn ceramic balls sliding with the three kinds of samples (SS, TD150 and TD150 with solid lubricants) at different speeds are presented in Figure 9. It can be seen that the wear volumes continue to increase as the sliding speed changes from 20 m/min to 100 m/min for all the samples. Under the same test condition, the TD150 sample arouses a lower wear loss of paired ceramic ball compared to that for the SS sample, while the TD150 sample with solid lubricants arouses the lowest wear loss.

**Figure 10:**
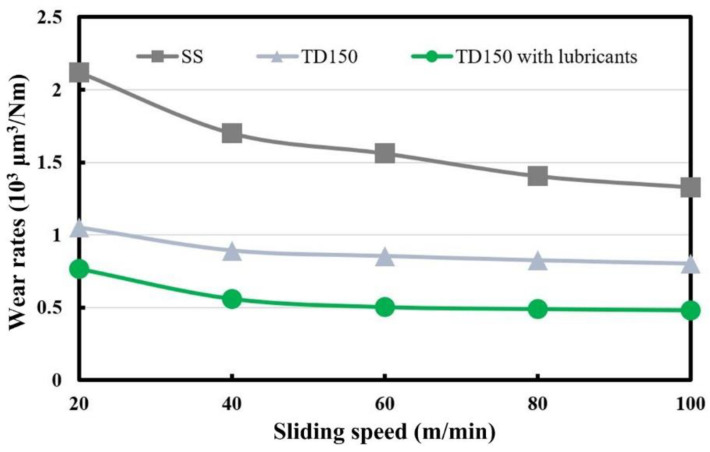
Wear rates of the worn ceramic balls sliding with different samples at different speeds.

Wear rate can be defined as the volume loss divided by the sliding distance multiplied by the applied normal load. The wear rates of worn ceramic balls sliding with different samples at different speeds are calculated and presented in Figure 10. It can be seen that the wear rates of worn ceramic balls continue to decline as the sliding speed changes from 20 m/min to 100 m/min for all the samples. Under the same test condition, the TD150 sample arouses a lower wear rate of the paired ceramic ball compared to that of the SS sample, while the TD150 sample with solid lubricants arouses the lowest wear rate. The calculation indicates that the wear rates of the ceramic balls can be reduced by 40~50% for TD150 and about 65% for TD150 with solid lubricants compared to that for the untextured one.

### 3.3. Worn Surface of the Cemented Carbide Disk

**Figure 11:**
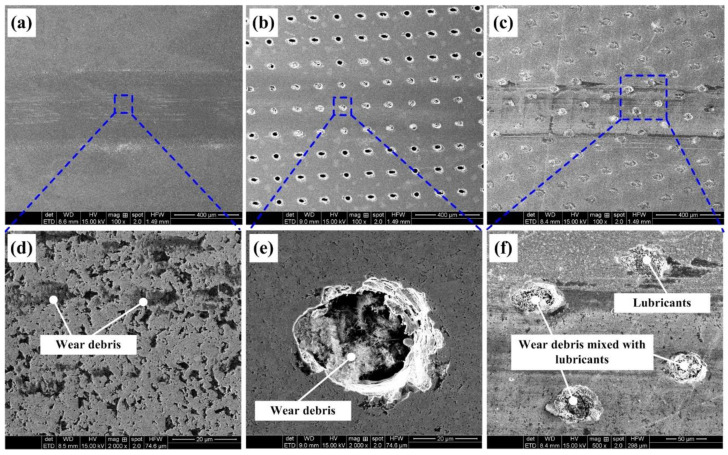
Wear tracks of different samples at sliding speed of 40 m/min: (**a**) SS, (**b**) TD150, (**c**) TD150 filled with lubricants, (**d**) enlarged view for SS, (**e**) enlarged view for TD150 and (**f**) enlarged view for TD150 filled with lubricants.

The wear tracks of different samples (SS, TD150 and TD150 with solid lubricants) at a sliding speed of 40 m/min are presented in Figure 11. It is obvious that the width of the worn track on the TD150 sample is narrower than that on the SS sample, while the width of the worn track on TD150 with solid lubricants is the minimum one. Residual wear debris can be found on the worn surface of the SS sample. However, there is no obvious wear debris on the worn surface of the TD150 sample and the debris is collected in the textured dimples. For the TD150 sample with solid lubricants, the textured dimples on the worn surface are filled with mixed components of wear debris and lubricants. In other words, the textured dimples have a combined effect of storing lubricants and capturing wear debris, which plays an important role in reducing friction.

### 3.4. Discussion

**Figure 12:**
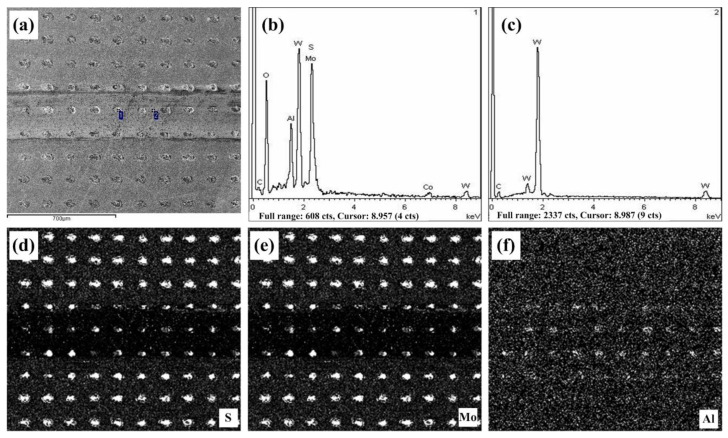
Worn surface and component analysis for TD150 filled with lubricants at sliding speed of 40 m/min: (**a**) SEM micrograph, (**b**) XRD for point 1, (**c**) XRD for point 2, (**d**) EDS map of S element, (**e**) EDS map of Mo element and (**f**) EDS map of S element.

The worn surface and component analysis for the TD150 sample filled with lubricants at a sliding speed of 40 m/min are presented in Figure 12. The XRD for worn dimple (see Figure 12b) shows the existence of element components of sulfur, molybdenum, tungsten, aluminum, oxygen, cobalt and carbon, which verifies the mixed ingredients of solid lubricants (MoS_2_) and wear debris (Al_2_O_3_ + WC + Co). The EDS maps (see Figure 12d–f) also prove the existence of mixed ingredients of lubricants and wear debris. In the present friction tests, abrasive wear is the main wear form for that the cemented carbide and the ceramic are typical hard brittle materials. Storing lubricants and capturing wear debris of textured dimples are both conducive to reducing abrasive wear. This is why the wear loss of the worn ceramic balls sliding with textured samples with or without lubricants is both lower than that sliding with the smooth one. Moreover, due to the combined effect of lubrication and debris capture, the texture sample filled with lubricants can lead to the minimum wear loss of the ceramic ball (see Figure 9).

It can also be seen from Figure 12d,e that the stock of lubricants is already low in the worn dimples compared to that in the non-worn dimples. In other words, solid lubricants are constantly consumed in sliding processes. This is exactly why the friction coefficient for the TD150 sample with lubricants is nearly equivalent to that for the TD150 sample without lubricants at the end of the sliding operation (see Figure 4). In other words, when the lubricants in the textured dimples are almost consumed, the TD150 with lubricants will not be able to provide sufficient lubrication continuously. However, in any case, the friction coefficient of the textured samples with or without lubricants is both smaller than that of the smooth sample. This can be attributed to the role of textured dimples in capturing wear debris and reducing friction. Textured dimples can not only catch wear debris but also cause friction vibration, which is attributed to a smaller friction coefficient with obvious fluctuation for TD150 without lubricants (see Figure 4). Obviously, the mechanism for TD150 improving friction is storing lubricants and capturing wear debris, which both lead to the reduction of friction coefficient and wear loss.

This work confirms the feasibility of surface texturing in improving the tribological properties of cemented carbide materials, and reveals the mechanism of textured dimples to improve friction, namely storing lubricants and capturing wear debris. The research work will play an important role in promoting the engineering application of surface texturing in cemented carbide materials.

## 4. Conclusions

Micro-dimples were fabricated on the surface of WC/Co cemented carbide disks by laser. Dry friction tests were carried out by sliding with Al_2_O_3_/WC ceramic balls. From the experimental results and discussion, the following conclusions can be drawn:

(1) Textured cemented carbide can reduce the average friction coefficient by about 30% compared to the smooth sample, while textured cemented carbide with solid lubricants can reduce the average friction coefficient by about 50%. The density of textured dimples has no obvious influence on the friction coefficient under the present conditions.
(2) The wear rates of worn ceramic balls continue to decline as the sliding speed changes from 20 m/min to 100 m/min for all the samples. The wear rates of the ceramic balls can be reduced by 40~50% for textured samples and approximately 65% for textured samples with solid lubricants compared to the untextured ones.
(3) The mechanism for improving the tribological properties of cemented carbide materials is that the textured dimples can store lubricants and capture wear debris, which would play an important role in promoting the engineering application of surface texturing in cemented carbide materials.

## Figures and Tables

**Table 1:** Properties of the WC/Co cemented carbide.

Composition (wt%)	Hardness (HRA)	Thermal Conductivity (W/m·k)	Thermal Expansion Coefficient (10^−6^/k)	Density (g/cm^3^)
WC +6%Co	91.0	79.6	4.8	15.2

**Table 2:** Properties of the Al_2_O_3_/WC ceramic.

Composition (wt%)	Hardness (HRA)	Thermal Conductivity (W/m·k)	Thermal Expansion Coefficient (10^−6^/k)	Density (g/cm^3^)
Al_2_O_3_ +35%WC +10%Co	89.0	45.2	3.5	10.3
